# The Bristol Meeting of the British Medical Association, 1894

**Published:** 1894-09

**Authors:** 


THE BRISTOL

### Medico-Chirurgical Journal.

SEPTEMBER, 1894.

### THE BRISTOL MEETING OF THE BRITISH MEDICAL ASSOCIATION, 1894.

**The Birth of the Association—The 1833 Meeting in Bristol—Bristol's Part in the Association work from 1834 to 1862—The 1863 Meeting in Bristol—1864-1893—The Preparation for the 1894 Meeting in Bristol—The Work of the Committees:** *Finance; Printing and Publishing; Reception; General Purposes; Pathological Museum; Local Entertainments; Dinner and Luncheon; Excursions; Annual Museum; Executive*—**The Daily Journal—The General Meetings—The Cathedral Service—The General Addresses—The Sections:** *Medicine; Surgery; Obstetric Medicine and Gynæcology; Public Medicine; Psychology; Pathology; Ophthalmology; Laryngology and Otology; Dermatology; Diseases of Children*—**The Pathological Museum—The Annual Museum—The Work at the Hospitals—The Day Entertainments:** *Visits to Factories; Medical Missions Breakfast; Luncheon at Clifton Spa; The Garden Parties; Medical Temperance Breakfast; Luncheon at the Merchant Venturers' Hall; Organ Recital; Inspection of "Old Bristol"; The Mayoress's "At Home"*—**The Evening Entertainments:** *Conversazione by Museum Exhibitors; The President's Reception; Public Dinner; Smoking Concert; The Mayor's Concert and Ball*—**The Short Excursions:** *The Docks; Bath; Clevedon; The Water Works*—**The Long Excursions:** *Berkeley and Thornbury; Glastonbury and Wells; Weston-super-Mare and Cheddar; Ilfracombe; Chepstow, Tintern, and Symonds Yat*—**General Summary.**

THE recent coming of the British Medical Association to Bristol naturally carries the thoughts to the other meetings of the Association which have been held here, and to the share taken by Bristol men in advancing the professional work of the Association and shaping its policy.

### THE BIRTH OF THE ASSOCIATION.

On July 19th, 1832, about fifty doctors met in the Worcester Infirmary and formed the Provincial Medical and Surgical

Association, which a hundred and forty medical men had declared themselves ready to join. Bristol was represented at the meeting by Mr. William Hetling. The objects of the Association are not defined in the resolutions passed at that meeting, but are outlined in the introductory address which was then delivered by Dr. Charles Hastings, to whose exertions the Association owed its existence. In the light of after-events, it is interesting to note that the new Society came into existence with the anticipatory blessing of the *Lancet*, which on July 7th, 1832, said that the scheme laid "a foundation for the congenial union of scientific research and professional honour." As its name implied, its sphere of action was to be confined to the provinces. If the annual address consisted of a biographical memoir, the hero whose praises were to be said or sung and whose work was to be thus immortalised must have resided in the provinces. The President was to be a physician or surgeon of eminence dwelling in one of the provincial towns visited by the Association. The Council was to be selected from the doctors of the principal provincial towns. Matters have changed since then, and London doctors should be grateful that they have been allowed to join an Association which, with a continuous history, although with a changed title, has become the most powerful Medical Association ever known on this globe.

At the Worcester meeting, Dr. Andrew Carrick,[1] then senior physician to the Bristol Infirmary, was appointed President-elect. A Council was formed, which included Henry Clark,[2] Lecturer on Anatomy at the Bristol Medical School; Dr. Henry Hawes Fox,[3] who had been physician to the Bristol Infirmary; William Hetling,[4] surgeon to the Infirmary, and Lecturer on Surgery at the School; W. F. Morgan,[5] house surgeon at the Infirmary; William Mortimer, a surgeon in practice at Clifton; Dr. Henry Riley,[6] Lecturer on Medicine and Anatomy at the School; Richard Smith,[7] then senior surgeon to the Infirmary;

[1] *Bristol M.-Chir. J.*, vol. viii., 1890, p. 170.
[2] *Ibid*, vol. x., 1892, pp. 267, 286. [3] *Ibid*, vol. viii., 1890, p. 174.
[4] *Ibid*, vol. viii., 1890, p. 182; vol. x., 1892, p. 268.
[5] *Ibid*, vol. viii., 1890, p. 170.
[6] *Ibid*, vol. viii., 1890, p. 168; vol. x., 1892, pp. 267, 287.
[7] *Ibid*, vol. viii., 1890, p. 179.

and Dr. George Wallis,[1] physician to the Infirmary and Lecturer on Anatomy.[2] The other Bristol men who joined in the first year were Charles Anthony, Dr. Charles E. Bernard, Dr. George G. Bompass, W. O. Cleave, Robert Corbin, Henry Daniel,[3] surgeon to the Infirmary, Dr. David Davies, senior surgeon to St. Peter's Hospital, John Bishop Estlin, William Falls, Drs. Edward Long Fox, Charles Joseph Fox, and Francis Ker Fox, of Brislington, Edward Gee, W. J. Goodeve, Thomas Green,[4] Dr. Thomas Griffiths, Edward Halse, Josiah Hill, Joseph J. Kelson, John King, Joseph G. Lansdown, Robert Lax, Isaac Leonard, Dr. Gilbert Lyon, physician to St. Peter's Hospital, Dr. W. Ogilvie Porter, Richard Powell, Dr. James Cowles Prichard,[5] physician to the Infirmary, James Prowse, Thomas Roblyn, surgeon to the Clifton Dispensary, Charles Smerdon, Nathaniel Smith,[6] surgeon to the Infirmary, T. L. Surrage, John C. Swayne,[7] Lecturer on Midwifery at the School, Dr. J. A. Symonds,[8] physician to the General Hospital, and John Grant Wilson, surgeon to the General Hospital. Many of these shed lustre on local professional life, and altogether they formed an imposing collection of Bristol doctors.

In the first volume of the *Transactions*, William Hetling reported, with an illustration, a "Case of Osteo-sarcoma of both Jaws, in which Amputation at the Joint was Effected." The case is very fully described, but a large portion of the article (which occupies 63 pages) deals with the pathology of tumours of bone in general. A very full bibliography is appended to the paper. It is interesting to note the following paragraph, which shows some of the difficulties under which surgeons laboured in pre-anæsthetic days: "The operation was necessarily long and tedious, occupying about three-quarters of an hour, part of which time was spent in allowing the patient to rest and change her position, and in giving some cordial to relieve weariness, rather than actual state of fainting." On July 6th, 1833, the *Lancet* devoted more than ten pages to a summary and

[1] *Bristol M.-Chir. J.*, vol. viii., 1890, p. 168; vol. x., 1892, p. 265.
[2] At the School of Anatomy and Medicine in Limekiln Lane, now St. George's Road.
[3] *Bristol M.-Chir. J.*, vol. viii., 1890, p. 186. [4] *Ibid*, vol. x,, 1892, p. 272.
[5] *Ibid*, vol. viii., 1890, p. 176. [6] *Ibid*, vol. viii., 1890, p. 187.
[7] *Ibid*, vol. x., 1892, pp. 269, 289. [8] *Ibid*, vol. x., 1892 pp. 270, 290.

review of the volume, four papers from which it had printed in full, in an earlier number.

THE 1833 MEETING IN BRISTOL.

The first anniversary meeting of the Association was held at Bristol under the presidency of Dr. Carrick. The Council met at 11 a.m., on July 19th, 1833, in the Infirmary Committee Room, and at one o'clock the general meeting was held, at which nearly 200 members were present. Dr. Carrick opened the meeting with a speech which does not appear in the *Transactions*, but which was printed separately and circulated among the members, who then numbered 316. The address was described as "luminous" by *Felix Farley's Bristol Journal* of Saturday, July 20th, whose report of the meeting occupies a page and three-quarters in the *Lancet* of the 27th. This does not include Dr. Carrick's Address, which appears, copied from the *Bristol Mirror*, in the next issue of the *Lancet*, which spoke of it as reflecting the highest credit on his feelings and judgment. The Retrospective Address was given by Dr. Edward Barlow, of Bath. Of the Bristol doctors who had become members by that time John Colthurst[1] and George Hillhouse Hetling[2] are still living. Several plans were adopted for the future. One of these was the appointment of foreign correspondents who should put themselves into communication with eminent physicians and surgeons in other countries, with a view of obtaining a record of medical work. Dr. Prichard was appointed for Germany, but seems, from a note in the Report of the following year, to have declined the post. No cases or papers were read. The meeting occupied only one day. The festivities of the occasion are not reported in the *Transactions*, but from what we know of the habits of some of the members and the custom of the period, there can be little doubt that, in the limited time at disposal, they were duly observed. We learn from other sources that the company dined at six o'clock, at Ivatts' hotel.[3] Dr. Carrick, of course,

[1] *Bristol M.-Chir. J.*, vol. x., 1892, p. 272.

[2] *Ibid*, vol. viii., 1890, p. 183; vol. x., 1892, p. 272.

[3] This was the Royal Gloucester Hotel, at the Hotwells, which stood on the ground now occupied by Lady Haberfield's Almshouse.

was in the chair. Mr. Richard Smith and Mr. Hetling were the vice-presidents. "Nothing could exceed the harmony and conviviality of the party. . . . . The company did not rise from this social and intellectual entertainment until a late hour. . . . . There never has been, on any occasion, such an assemblage of provincial talent and character convened together in this or any other country." The turtle, dessert, wine, and every arrangement respecting the dinner are said to have done Mr. Ivatts the highest credit. The toast-list was a comprehensive one, as may be seen from part of it printed in *Felix Farley* of July 27th, which also contains a report of Dr. Carrick's speech at the annual meeting. A melancholy incident occurred at the close of the meeting. One of the members, a much esteemed and most exemplary retired surgeon from the country who had been at the dinner, at which he had taken two glasses only of wine, and had afterwards wound up his watch, was found dead in bed in the hotel when the chambermaid went to call him in the morning.

The *Lancet* had an editorial on the meeting taking exception to the title of the Society, and suggesting "British Medical Association," a name which was afterwards adopted by a Society formed in 1836. It expected that immense advantages would arise from the establishment of this Association, which it urged to direct its attention to the subject of an efficient medical reform.

### BRISTOL'S PART IN THE ASSOCIATION WORK FROM 1834 TO 1862.

In the second volume of *Transactions* is a contribution by Drs. Carrick and Symonds on "The Medical Topography of Bristol." Typhus and typhoid had not then been differentiated. The authors, referring to cases of continued fever, say that nine-tenths are of a simple character, but cases which are longer in their duration "have inflammatory complications, which render the terms cephalic, bronchitic, gastro-enteric appropriate adjuncts." They note that "in the *post-mortem* examinations of febrile cases, ulceration of the mucous membrane of the bowels is the most common morbid appearance." An interesting article is concluded by an earnest appeal for the registration of deaths in the places where these occur, instead of at the place of burial. Also, in the second volume are contributions by Thomas Green on "The Treatment of Syphilis without Mercury," by W. F. Morgan on "Dislocation of the Shoulder," and "A Description of the Anatomical Structure of the Liver of a Rat, from Cuba," by Dr. Henry Riley.

The third volume of *Transactions* contains two communications from Dr. Symonds. One is on "The Progress and Causes of Cholera, as it occurred in Bristol in 1832." The other is a very full report of the case of Mrs. Smith,[1] whose death from arsenic poisoning was proved by the exhumation and examination of the body fourteen months after death. Dr. Riley, Mr. Kelson, and Mr. William Herapath, who was then Lecturer on Chemistry and Toxicology at the School, were officially associated with Dr. Symonds in the investigation. The crime was brought home to Mrs. Burdock, who was hanged on April 15th, 1835. Dr. Symonds's paper is accompanied by a coloured illustration of the stomach and part of the intestines. The preparation itself was placed in the Medical School Museum.

At the 1835 meeting in Oxford the retrospective address was given by Dr. J. C. Prichard, on whom the University then conferred the degree of M.D. by diploma. The address is printed in the fourth volume of the *Transactions*, in which Bristol was also represented by a communication from Dr. Paris Thomas Dick, on "The Unity of Organic Structure," which was continued in Vol. V.

When the Association went to Bath in 1838 the retrospective address chronicled the deaths of Carrick and William Hetling.

At the meeting at Liverpool in 1839 Dr. Symonds delivered the retrospective address, which covers 97 pages of the *Transactions*. At this meeting a "Report on the Progress of Surgery" was given for the first time.

At the York meeting in 1841 the Council stated in their report that they were working out a plan by which each member of the Association, without additional subscription, should

[1] *Bristol M.-Chir. J.*, vol. x., 1893, p. 268.

receive the *Provincial Medical and Surgical Journal*, a weekly periodical which had been started on October 3rd, 1840. A law to that effect was soon after added to the Code of the Association.

When the Association met at Exeter in 1842 Dr. William Budd exhibited and explained at some length a number of casts and drawings that had formed the groundwork of the paper, "On Diseases which affect Corresponding Parts of the Body in a Symmetrical Manner," which, in December, 1841, he had communicated to the Royal Medical and Chirurgical Society of London.

Volume XI. (1843) of the *Transactions* contains a record by Dr. George Wallis of "Some cases showing the Advantage of Powerful Counter-irritation, especially the Long Issue on the Calvarium."

In 1844, at the Northampton meeting, Dr. Budd, in the place of Dr. Riley, who had been appointed to do it at the previous meeting, gave a retrospective address on "Anatomy and Physiology." This occupied nearly three hours in delivery. It is published in Vol. XIII. of the *Transactions*. Apparently unexhausted by his efforts of the night before, Dr. Budd, at the meeting of the second day, when it was proposed for Journal purposes to increase the subscription, carried an amendment appointing a Committee to take into consideration whether it be expedient to continue the publication of a weekly medical periodical. He considered that the multiplication of such periodicals was an obstacle to the profession.

At the Sheffield meeting in 1845 came a report from this committee, a meeting of which had been held in Bristol, when eight of its members were present; but they did not arrive at any final determination. As Dr. Budd was not able to attend, the presentation of the report was deferred.

At the 1848 meeting at Bath, Dr. Budd carried a resolution protesting against the regulation of the College of Surgeons which had laid down that candidates for the Fellowship should have three years of their student life passed in London.

It would seem that by this time Dr. Budd had achieved a good fighting reputation, for in July, 1849, he was deputed by the Bath and Bristol Branch to go to the annual meeting at Worcester in the following August in order to enforce the views of the Branch about securing for the *Journal* an editor of first-rate talent in the place of Dr. Streeten, who had died in the previous May.

In the 1850 volume of *Transactions*, Mr. Hore, the house-surgeon at the Infirmary, published a "Report of the Medical and Surgical Cases treated in the Wards of the Bristol Infirmary during the years 1848 and 1849."

When the Association met at Swansea in 1853 the "Retrospective Address in Surgery" was given by Mr. Augustin Prichard. This, according to the usual practice, would have been printed in the *Transactions;* but as the *Journal* absorbed so much money, the publishing of the annual volume was deferred, and in the end no more volumes of *Transactions* were issued. Thus the Surgical address and others did not appear in the records of the Association. There followed much controversy upon this, and in the *Journal* of November 17th, 1854, is a letter from Mr. Prichard on the subject.

At the Manchester meeting in 1854 Dr. J. G. Davey read a paper on the "Law of Lunacy."

The 1855 meeting at York found the Association in great turmoil. There was much fault found with the management, editorial and otherwise. A great crisis had been reached, and the Association seemed in peril of death. On September 20th Dr. Budd, at a meeting of the Bath and Bristol Branch, of which he was President, made an eloquent appeal to those who were leaving the Association that they should stand by it. As a result of this, a special meeting of the branch was called, when, upon the proposition of Dr. Davies, seconded by Mr. R. W. Coe, it was resolved to approach the movers of the secession party, with a view to retaining them in the Association. The members were summoned to a general meeting at Birmingham on November 20th, when there were present from Bristol, Dr. Budd, Mr. Coe, Mr. Crosby Leonard, Mr. W. F. Morgan, Dr. J. O'Bryen, and Mr. S. H. Swayne. Such a conciliatory spirit was shown by everyone that the meeting "swept away every element of discord within the bosom of the Association, and in the bursts of cheering with which this happy accord was greeted every member present felt that the Association was taking a new and lengthened lease of its existence."

In 1856 the annual meeting was held at Birmingham, when, with very slight alterations, the resolutions of the special meeting became the new laws of the Association. The Association changed its name to the British Medical Association, as the old society with that title, the main object of which was to push on questions of medical reform, was dead. At Birmingham Dr. Budd was well to the front on the questions of medical reform and the diffusion of cholera by means of water. In reference to the latter, he paid a tribute to the prior investigations of Dr. Snow. At the dinner, in proposing "The Birmingham and Midland Counties Branch," he made a speech which was loudly applauded.

At Nottingham, where the annual meeting was held in 1857, Dr. Budd proposed a resolution, which was carried unanimously, to expel one of the vice-presidents, who had become a homœopathist. Dr. Budd said that, of all forms of quackery, none was so insidious, so miserable and wretched, as that form of it called homœopathy. He was again very emphatic on the subject of medical reform.

At the Torquay meeting in 1860 the name of a Bristol surgeon was ordered to be struck out, in consequence of his having avowed himself a homœopathist. In a discussion on the question of special hospitals, Dr. Budd protested against the establishment of such for diseases of the nervous centres. He also appealed for the use of photography in medicine, and showed some photographs of diseased structures taken by himself. Dr. Davey spoke on the question of representation of the profession in Parliament, and took part in a discussion on the suggestion of forming a Medical Diaconate, which the Rev. Chancellor Martin brought forward at the request of the Bishop of Exeter.

In 1862 the Association went for the first time to London. Opportunities for scientific triumphs were great. The Royal Colleges made special efforts to make the meeting a social success. As metropolitan reputation was at stake private hospitality, no doubt, was profuse. It was therefore courageous on the part of Bristol to be willing to take the meeting in the year immediately succeeding. Drs. Budd, Burder and Davey, Messrs. Green and Lancaster, and Dr. Henry Marshall were at the London meeting; and on the proposition of Dr. Waters, of Liverpool, seconded by Dr. Budd, the invitation of Bristol was accepted.

THE 1863 MEETING IN BRISTOL.

In the *Journal* of May 2nd, 1863, there is a short notice of the coming meeting. On June 18th Dr. Francis Ker Fox, in his Presidential address to the Bath and Bristol Branch, thus alluded to it: "The annual meeting of the parent Association draws near; and, when we recollect the philosophical character of the addresses which were read at the gathering last year, as well as the enlarged hospitality which provincial members received from their metropolitan brethren, the Bristol Branch may be excused for feeling some little distrust of its power to follow such a bright example. The Addresses in Medicine and Surgery have, however, been allotted to men not unknown to fame, whose frequent contributions to science afford the best guarantee that these gentlemen will reflect credit upon the selection of the Council, and who possess this especial recommendation that each of them, Dr. W. Budd and Mr. Prichard, are men of eminently practical minds. And, although the subjects of obstetrics and of chemistry will probably attract the attention of a more special audience, we feel assured that the authors of those addresses will illustrate their several papers with every novelty which recent scientific researches may have brought to light; that the more frequent practice of ovariotomy and the unlimited application of the speculum will not escape the notice of Dr. Swayne, and that the practice of medical chemistry will be sanctioned and advanced in the address of Dr. Herapath. Our highest expectations, however, are built upon our intimate knowledge of the mental resources of our learned President, of whom we may truly say that, although he be a provincial physician, he is *haud ulli secundus;* and, I may add without flattering, that this Branch reposes perfect confidence in the philosophical and enlightened character of his address, and in the enlarged hospitality with which he proposes to inaugurate the meeting." At the Branch Annual Meeting the only other reported allusion to the approaching visit is this paragraph in the Council's report: "The annual meeting of the parent Association having been announced to be this year held in Bristol, on the 5th, 6th and 7th of August, the Council beg to express a hope that the members of this Branch will cordially unite in doing all that lies in their power to render the meeting successful and agreeable." If the grammar of these communications cannot be commended, yet it must be admitted that their good intentions were admirable. On the first three Saturdays in July the *Journal* gave a notice of the meeting fuller than that in May, and on the last Saturday this was added: "The 'Queen's' (situated close to the Victoria Rooms), the 'Bath' (Clifton), and the 'White Lion' (Bristol) are among the best hotels. A clerk will be in constant attendance at the Victoria Rooms during the days of the meeting, and will give information regarding lodging-houses, which are numerous, and where beds may be had from two shillings a night upwards." In the issue of August 1st the titles of papers promised were added to the writers' names which alone had previously been announced.

On Wednesday, August 5th, the work began with a meeting of the Committee of Council at 1 p.m. On this and the other days of the meeting the local doctors supplied the visitors with lunch in the Victoria Rooms. At 2.30 the General Council met. At 4, in the Victoria Rooms, the first general meeting was held. 195 members had entered their names. It is estimated that, as many omitted to record their names, there were nearly 250 present altogether. Dr. Burrows, the President of the past year, said a few words of farewell, and then resigned the chair to Dr. Symonds, who at once delivered the Presidential Address. The General Secretary then read the Report of the Council. The remainder of the meeting was occupied with the election of officers, voting of thanks, and passing a resolution to form a committee for reporting on the question of a Medical Provident Association for the benefit of doctors and their families. In the evening at 9 there was a conversazione at the

Philosophical Institution in Park Street. There seem to have been no ladies present, although it is reported that the company at the Wednesday soirée at Canterbury in 1861 included many of that sex. The entertainment was mainly scientific. Electrical experiments were shown by Mr. Phillips, of Weston-super-Mare. Mr. W. Sanders discoursed on the "Geological Peculiarities of the District." There was a kind of pathological museum on view, for which the meeting was indebted to Dr. Samuel Martyn and other local doctors, and to Dr. Lionel Beale. To give a lighter tone to the assembly, the civic plate was lent by the Mayor, Mr. Sholto Hare, and supper[1] was served at 11. A little after 12 the entertainment ended.

On Thursday, the General Council met at 11. At noon the second general meeting was held. After some business matters had been transacted, the Address in Medicine was delivered by Dr. Budd, who chose for his subject, "The Laws of Contagious Epidemics." At 3.30 the meeting was resumed. The Address in Surgery was given by Mr. Augustin Prichard, whose topic was "Carbuncle." At 9 the President gave a reception at his residence. For the amusement of his guests he had provided a band and a company of glee-singers. The gardens were illuminated, and the lime-light exhibited by Mr. Phillips as a great novelty. "A sumptuous repast" was served at 11, and soon afterwards the party dispersed.

On Friday at noon there was a general meeting, at which Reports were received and an Address on "Chemistry in its Relation to Medicine and its Collateral Sciences," was delivered by Dr. W. Bird Herapath. At 3.30, Dr. J. G. Swayne gave the Address in "Midwifery," which, as it referred only to points in midwifery practice about which there were no very clearly defined rules, disappointed Dr. Francis Fox's expectations

[1] A contributor to the *Bristol Times* said : "I never saw people eat with so great a contempt for the nightmare as did the professional part of the company. . . . Everything you could conceive unwholesome to retire to bed upon, from lobster salad to pork pie, was swept down before the repeated charges of these dyspepsia-defying *lancers*. I jogged the elbow of my medical attendant just as he went in for a third helping of raised pie, half a plateful of which would have made me dream that I had the Observatory on, or a plesiosaurus in my stomach. 'Pray remember the poor inside,' I said. 'See if you can reach that decanter of sherry for me,' he merely replied, with his mouth half-full."

about the speculum. Papers and cases followed this, as they did all the other addresses, and the usual votes of thanks concluded the work of the meeting. The dinner, at which 214 were present, took place in the small room of the Victoria Rooms. Dr. Symonds presided, and the vice-chairs were occupied by Dr. Budd and Dr. Marshall. The proceedings at the dinner were somewhat marred by an episode which arose out of a misunderstanding in reference to one of the general addresses. Some statements concerning it were afterwards made in one of the local newspapers.

The *Journal* afterwards spoke in most laudatory terms of the meeting from both the scientific and the social standpoints.

The *Medical Times and Gazette*, a candid critic of the Association's methods and results, spoke of Dr. Symonds's address as one of those scholarlike essays that "come before the world stamped with the authority of cultivated and unbiassed minds." The same critic was impressed with the "high degree of originality and appropriateness" of Dr. Budd's address, and alluded to "the eloquent manner of its delivery." The address in Surgery was said to be "that of a sound practical surgeon," and that in Midwifery to be the production of "one who sees with his own eyes, and who, as he can trust himself, is worthy to be trusted by others." In an impartial article it summed up the results of the meeting by saying that "those who had the good fortune to be at the gathering will long retain cheerful recollections of days pleasantly as well as profitably spent."

At that time the acerbities of medical journalism were very pronounced. The *Lancet*, between which and the Association *Journal* there had been many a conflict in forcible language, thought nine lines ample for a report of the Bristol meeting. It printed neither of the addresses nor any of the papers.

### 1864—1893.

The proceedings of the Association during this period are so well known and information about it is so easily accessible, that it is not necessary to give a recapitulation in detail; but of the recent meeting it is desirable to place on record a connected account, taking notice of the numerous ramifications of this large and important Association. The work of future meetings will also be facilitated by mentioning errors which may be corrected, and alterations which may with advantage be made. Of late years steps of a more or less decided character had been taken more than once to invite the Association again to Bristol; but, for various reasons, postponement was considered desirable, and it was not till the 1893 meeting at Newcastle-on-Tyne that the formal invitation was given. It was at once accepted.

### THE PREPARATION FOR THE 1894 MEETING IN BRISTOL.

In accordance with ordinary but somewhat unsatisfactory practice, six local doctors were appointed to meet the same number of the Council and carry out the preliminary arrangements. The Bristol men were Mr. Nelson C. Dobson, Dr. E. Long Fox (who was to be the President), Mr. W. H. Harsant, Dr. Henry Marshall (who had been Local Secretary at the 1863 Meeting), Dr. E. Markham Skerritt and Dr. R. Shingleton Smith, the two latter respectively the Bristol Secretary and the President of Bath and Bristol Branch.

The Council, on the recommendation of the Committee of Management, chose orators for Addresses in Medicine, Surgery, and Public Medicine, and also appointed the officers of the Sections in which the scientific work of the Association was to be done. At the beginning of 1894 the local members of the Association were summoned, and, at a meeting held in the Medical Library, formed the various committees to which was entrusted the carrying out of matters outside the work of the Scientific Sections.

### THE WORK OF THE COMMITTEES.

The members of the various committees entered with great cordiality into the work assigned to them, and after-events clearly proved that no better men could have been found for their posts than the Secretaries, upon whom most of the labour fell, and who seem to have been chosen by a happy inspiration.

*The Finance Committee.*—The collection of the money, now necessarily a large sum, for meeting the expense of a visit of the

Association was at first an anxiety. The first circular which the Committee issued asked for unconditional donations, and expressed a hope that the money would be paid at once. The error of not asking simply for a guarantee fund, with a promise of a *pro ratâ* return of unused money, was afterwards corrected, and eventually there was no difficulty in obtaining the requisite promises.

A place like Bristol, with a large medical population, is not distressed by the question of expense; but certainly the time has now more than arrived when the Central Association, with its large profits, should provide most of the cost of its Annual Meeting. If this is not done, the smaller towns will find it impossible to receive the Association, with its largely-increased roll of members. The second of the "Regulations for the Conduct of Annual Meetings of the British Medical Association" tells the medical men of the locality visited that they are not to deem it necessary to incur a large expenditure. No town will wish to be considered more shabby than its predecessor, and therefore a large expenditure is necessary. This regulation should be revised. The Central Association might make contributions to the expenses of the meeting in proportion to the population of the town visited.

*The Printing and Publishing Committee.*—The main business of this Committee is to attend to the wants of the other Committees, over which it has to keep a very watchful eye, to see that they do not incur extravagant or unnecessary expenditure. Amongst many duties of a minor character, in the way of supplying stationery and placards and notices for the easy finding of places of meeting, there is, however, one important matter, which in every town should have attention, and that is the provision of a local guide. It is a great comfort and welcome to a member getting to a place strange to him, to have put into his hands directly he arrives a convenient and trustworthy guide-book. It makes him in a certain sense feel at home at once. This year, owing to the enterprise of Mr. James Baker, of Clifton, the Committee had no difficulty in this matter, as he placed at their disposal a thousand copies of a pleasantly-written guide, which he edited for the occasion, and of which he wrote a considerable portion. It was a pity that he allowed the outside of this attractive book to be disfigured by obtrusive advertisements; for when first received, it was difficult to be certain of its nature, as announcements of foods and drinks, wall paper, and cocoa almost overpowered its title. But the inside was better, and decidedly worth reading. Its illustrations were weak, especially the reproductions of photographs, some of which were really grotesque. But the generosity of the donor deserves much praise. We must not look a gift-book on the cover. A great deal of the expense with which this Committee deals should be defrayed by the Central Association, which ought to pay for all the printing expenses of the Pathological Museum and those connected with the notices of the rooms, and the writing materials supplied for them.

*The Reception Committee.*—Much of the comfort and smooth working of the meeting depends upon the work of this Committee. The allotment of the rooms for sectional purposes will perhaps never give universal satisfaction. In the opinion of their officers some sections will always have an undue importance. Upon this occasion, however, they were all fairly well accommodated. The rooms for Dermatology, and for Diseases of Children, holding only forty seats each, were rather small. In the former some members had to stand on Thursday morning, and possibly some went away when they found there was lack of room. The Ophthalmology and Laryngology rooms, with sixty seats in each, did perfectly well. The other sectional rooms each held comfortably at least 100, and were quite satisfactory. The large Victoria Room was used for the General Meetings and two of the three addresses, and was admirable for the purpose. It would be an advantage at future meetings if all the rooms had some floor covering to lessen the noise of the coming and going of members whilst the proceedings are being transacted.

The Council Room and General Secretary's Room were admirably placed near the large hall where the General Meetings were held. Commodious reading and smoking rooms were provided both at the Victoria Rooms and at University College. The ladies had also a good room in the latter building. At future meetings a committee of resident ladies should be formed to look after the interests of lady visitors, many of whom are now doctors. Relays of two members of this committee should be always in the ladies' room.

The small Victoria Room made an excellent Reception Room; there was plenty of space to move about in it. It was a wise thing not to furnish it too luxuriously, as no encouragement should be offered to members to linger therein. Two clerks from headquarters, who were accustomed to the work, drilled the hired clerks in their duties, and one of them was always present to superintend the work and to give any information that might be required. It would be an advantage if more of such clerks were sent to the Annual Meeting, as the work is entirely new to the local assistants, and unnecessary delay is thereby caused, more particularly at the beginning of the week when the rush for tickets of all sorts is great.

Similar remarks apply to the Post Office. This was most admirably placed just within the entrance to the Victoria Rooms. A permanent clerk knowing exactly what is required would be of great service. Part of his work should be to send on to members' local addresses any letters that had not been applied for at the end of the day. The messengers whom it is necessary to keep near the Reception Room, and who should be men rather than boys, could be utilised for this purpose. The plans of the rooms were excellent. Those of University College were kindly provided by Mr. Bond, the architect. That of the Victoria Rooms was specially drawn for the occasion.

It would be an advantage if the Reception Room could be kept open on the second day till 9 p.m. Many members come to the meeting only to attend the sections. Arriving late on that day they cannot even get the Daily Journal. The *British Medical Journal* of the week before the meeting stated the time when the Reception Room would be opened, but said nothing about the closing hour.

*The General Purposes, Hotels and Lodgings Committee.*—The duty of providing private accommodation for guests, which was undertaken by this Committee, is one that should be very rarely performed. Efforts should certainly be made to secure such accommodation for distinguished foreign and English doctors, many of whom are specially invited to the meeting, for representatives of Indian and Colonial Branches, and, perhaps, for Officers of Sections, but the provision of private residence for ordinary members, some of whom may be desirous of saving hotel or lodgings bills, should be discouraged. A Medical Association differs from many scientific gatherings. A large part of each day is, or should be, occupied by the guest with his professional work, in which his host or hostess cannot join, and so it is often merely offering bed and breakfast to one whom there is little opportunity of meeting socially. The printed circular which the Committee sent to a large number of residents was not calculated to add to the dignity of the meeting. The recorded results on this occasion were, no doubt, good, but the Committee would not be likely to hear any unfavourable reports. Cordial thanks are due to those residents of the neighbourhood who strove to make this social part of the meeting pass off satisfactorily. The list of available lodgings was larger than has ever been published before, owing partly to the fact that in Clifton there is so much accommodation of this kind, and partly to the systematic and complete way in which the work of the Committee was done. A district was apportioned to each member, and suitable houses were inspected. In doing this the married members of the Committee were very efficiently helped by their wives.

*The Pathological Museum Committee.*—In making its preparations for the Pathological Museum, this Committee was actuated almost entirely by the desire to exhibit only rare specimens or those illustrating certain groups of disease. Circulars were sent to members of the staff of all the principal hospitals in the neighbouring counties asking for such contributions. Little response, except from the Bristol hospitals, was made to these, possibly because it was so distinctly stated that only rare specimens would be welcomed. Letters were sent to the readers of papers in the Pathological Section suggesting that their illustrations, microscopic and otherwise, should be placed in the Museum, for which a large contribution was thus obtained.

*The Local Entertainments Committee.*—The greatest courtesy was received from the numerous persons with whom this Committee was brought into contact. The arrangements for visiting factories involved a considerable correspondence and many personal visits, and the result was eminently satisfactory, as a long list of these was offered to the members and their ladies. In relation to the Garden Parties and the short excursions that came within the jurisdiction of this Committee, much tact and discrimination were required. It is a matter for regret that members did not avail themselves of the opportunity of seeing the lovely scenery of the neighbourhood by the drives which had taken so much trouble to arrange.

*The Dinner and Luncheon Committee.*—Amongst the work of preparation which falls to the lot of this Committee is the anxious one of obtaining a suitable *menu* which a responsible and trustworthy caterer is prepared to carry into practical effect at the price named. The present arrangement of compiling the toast-list by the joint action of the Council of the Association and the local Committee is unsatisfactory, and should in future be left entirely to one only of these bodies; the Annual Dinner, according to existing regulations, is under the control of the Council. The decoration of the room in which the dinner was to be held was a matter to which the Committee gave careful consideration. One of the duties of the Committee as stated in the official suggested Regulations is: "To provide a sufficient number of vehicles for the conveyance of members home after dinner." This was well attended to, but the members seem to have preferred their own way of getting home. It falls within the business of this Committee to make arrangements for Daily Luncheons at moderate prices. This is less hospitable than the practice of the 1863 meeting, but it is nevertheless a great convenience to many members. It is essential that the Luncheons should be near the Sections, and, as there was no large room available, a suitable tent was provided for the purpose in the grounds of University College.

*The Excursions Committee.*—The work before this Committee was one of much responsibility. To provide a sufficient number of day excursions, and not too many, must be always a matter of great difficulty. After much deliberation it was decided to make arrangements for five. Entertainments by the nobility who are able to show historic houses, with their rich and varied contents, are always popular. Facilities, however, for these in this neighbourhood are few, and for four of their excursions the Committee had to trust mainly to the attractions of magnificent scenery and objects of a more or less antiquarian interest, visits to which are easy at any time. This, no doubt, was partly the reason why the arduous labours inseparable from arranging the numerous details connected with journey and refreshments were not rewarded by the success they deserved in attracting the number of persons for whom preparations were made. All the arrangements were excellently planned, an outcome of personal visits on many occasions by the Secretary of the Committee.

*The Annual Museum Committee.*—The extent of the details to be arranged by this Committee can be realised only by those who have been engaged in the work. Pre-eminently for them it would be desirable that the Association should provide an official manager who, familiar with previous necessities, would be able to carry on the work year by year, and take the initial steps requisite to the formation of each exhibition of drugs, instruments, foods, &c., which is now called by the inappropriate name of Museum. Under existing arrangements each secretary has to begin the work afresh. This is a distinct disadvantage, although he may possibly be helped, as was the case this year, by the courtesy of the preceding secretary. He cannot appeal to individual exhibitors of former meetings for advice about what is required, and how their interests are to be considered if the exhibition is to be a success. For this it might possibly be an assistance to him if out of those who are regular exhibitors at these meetings, a sort of committee of reference were appointed to which he might turn for opinions on some doubtful points. The exhibition grows year by year in importance and size, and it is unfair to a doctor in busy practice to throw upon him, as an entire novelty, the work of correspondence and detail required for the purposes of organisation. Even with this permanent manager there would still be room for the local secretary, who would find enough to do in looking after the carrying out of many of the details of the exhibition.

*The Executive Committee.*—This Committee consisted of the President-elect, the Local Treasurer, the Local Secretary (all of whom were members of all the Committees), the local members of the Management Committee, and the Chairmen and Secretaries of the other Committees. Its business was to receive reports from the other Committees, decide what should be done in reference to them, and take into consideration all subjects which did not come within the purview of those Committees. Everything was ready by Monday, July 30th, the opening day of the Reception Room.

### THE DAILY JOURNAL.

The Daily Journal is not so satisfactory as it ought to be. It has a "Local Editor," whose function is not to "edit" but to supply information of a general character, which is quite necessary, but could be easily obtained from ordinary commercial sources. Dual management, which seems to be a necessity for the Journal, is rarely satisfactory. Some alterations are required in printing the sectional information: for instance, it looks careless to print in one day's issue notices about discussions which have already taken place. One person should be responsible for seeing that this is not done, and for revising the "List of Members," in which many gross inaccuracies have usually occurred. A wealthy Association should not cumber the pages of its Daily Journal with advertisements.

### THE GENERAL MEETINGS.

It will probably be impossible to abolish the general meetings, but it is desirable that their powers should be considerably curtailed. A large number of members who attend them are not qualified to express opinions on the various technical reports that are brought before them, and which have received careful attention from a number of experts. Now that the Council is so thoroughly representative, much more authority should be given to it. It is sufficiently large to be free from party bias; and, far better than any general assembly, it can give calm and, if necessary, repeated attention to the questions that are submitted to it. Under present conditions, a snap division by a small number of members may hinder or prevent a much-needed reform, or settle off-hand a most intricate enquiry. It would be as reasonable to let a mass meeting of electors make the laws, which it is the business of the Houses of Parliament to do, as to continue the practice of allowing general meetings to affirm or negative the many abstruse propositions that are brought before them. The Council of the Association should be its real executive or governing body. If this were so, not only would business be more satisfactorily done, but the undignified scenes which are now enacted and reported in the newspapers would be impossible. It is now within the power of any buffoon or faddist, provided he is fluent of speech, to drag the Association through the mud, some of which it will be impossible to wipe off. Scenes such as we had at Bristol undoubtedly have their amusing aspect; but the Association exists not to provide fun, but for the serious advancement of our professional life. Editor-baiting, for instance, has always been a popular sport in the Association, and in the records of its proceedings many instances of it can be found. The editors have, however, usually been too much for their worriers. It is always easy enough to find fault, and an irresponsible person, ignorant of editorial difficulties, can without much trouble find flaws in the management. Perhaps the extreme of bathos in this direction was reached at this meeting, when an amusing person, finding fault with certain articles that had appeared in the *Journal*, dramatically threw to the floor the pages that contained them, as if he could no longer suffer himself to touch the unclean thing, then walked down from the platform and—carefully picked them all up. Some strong and influential leaders with tenacity of purpose are needed to grapple with this abuse of the general meetings, and save us from being, through the bad taste and misdirected zeal of a few, the laughing-stock of right-thinking persons.

### THE CATHEDRAL SERVICE.

It was unfortunate for the British Medical Association that the Cathedral Choir alterations which have waited so long for attention became so urgent in July that it was impossible to postpone them for another fortnight, because the members attending the service on Tuesday could see nothing of choir and chancel, but looking eastward were confronted with a barricading of rough boards. The Mayor and Corporation attended in state, and with the clergy and the members of the Council of the Association formed an imposing procession as they were marshalled to the seats reserved for them. The musical part of the service of course suffered through being accompanied by only a temporary organ. Those who had the arrangement of the nave had no doubt good reasons for placing the pulpit to the east of the temporary choir stalls, and so at the extreme end of the available space; but the result was that, while no doubt those in the transepts could hear the sermon well, to most of those in the nave it could have been little else but dumb show. Canon Ainger's sermon was an eloquent discourse on "The healing by the Saviour of the woman with the issue of blood," who had suffered more from the experiments of her physicians than from her malady. The preacher touched upon the prevalence of superstition and quackery both in religion and medicine, and commented at length on the unchangeable aims and methods of the true physician, whether of body or spirit, both being alike labourers in the Lord's vineyard.

Some of the members of the Executive Committee were appointed to see that seats were reserved for members of the Association and their friends. They were instructed to ask ladies who were not seen to be accompanied by a doctor if they were "with a medical man?" To this question one lady replied "I am one."

### THE GENERAL ADDRESSES.

This year all the addresses were distinguished more for excellence of manner than for quality of matter. If Association orations are not to be of some considerable solid value in the furtherance of our professional work, it is doubtful if much can be said for their continuance. The distinguished men who are chosen to deliver these addresses should set themselves stedfastly to accomplish something more than the tickling of the ears of four or five hundred medical practitioners who have been drawn away from pleasant pursuits by the attractions of a great name; but who, after they have pondered over that which they have heard, find difficulty in seeing what addition has been made to the store of medical knowledge. If better results than we have had lately are not possible, it will be wise to return to the early practice of the Association and have addresses which do not profess to be anything more than careful and convenient summaries of the gain which has been achieved by others in the previous twelve months, or else to devote the whole of the time of the meeting to sectional work, by which some fruit may be gathered.

*The President's Address* does not come quite within the scope of the foregoing observations. It is delivered by a man who has secured his position by local accident which no doubt often, perhaps generally, results in the best that can be obtained. No serious contribution is expected from him. The occasion is not one for the record of original research or for the delivery of a philosophical argument addressed to the higher intellectual activities with which the speaker is for the time brought into contact. The audience has upon some of these occasions had to strive to keep itself awake while the president at inordinate length has presented the history of his town from its origin, and of course exalted its virtues as a health-resort. But the meeting in Bristol was better served than this. For nearly an hour it listened with rapt attention and unfeigned pleasure to Dr. Edward Long Fox delivering, with much grace of utterance and of diction, a clear, practical, well-considered essay, in which he showed with no little eloquence and with more force of argument that the best and most useful lives in the world are those in which due attention is paid to the laws of physiology and hygiene, concerning which medical science has to be the exponent and the guide. With no lack of courage the President spoke words much needed now to prevent the triumph of the views of the well-intentioned but reckless fanatics who decry vaccination and the work of the Institute of Preventive Medicine. Passing also in review the gain accruing to the State from the increased medical attention paid to the feeble-minded and the poor, the Presidential

Address at the Bristol Meeting will be long remembered as, within its restricted limits, one of the best of its kind.

*The Address in Medicine.*—Much of a different sort is expected from the medical orator. His opportunity is a great one, and he must be judged by a different standard. By those who are qualified to judge, he is chosen on account of his professional eminence. On this occasion, in the person of Sir Thomas Grainger Stewart, due recognition was made not only of his individual qualifications but of the contributions which have been made to medical knowledge by the profession in Scotland, and by the celebrated Edinburgh School in particular. But Sir Grainger Stewart did not fulfil the expectations which had been formed of him and his subject. In attractiveness of delivery the address left nothing to be desired, and in itself it was by no means destitute of merit. It was almost a good post-graduate discourse. In reality it was a glorified clinical lecture, delivered under unusual conditions of environment and audience. Surrounded by his professional peers and faced by hundreds of attentive listeners, by many of whom he was considered almost an oracle, he failed to make the most of his opportunity, and dilated, fortunately with comparative brevity, on topics with which every observer of the recent influenza epidemics must have been more or less familiar. The orator admirably presented the existing knowledge concerning influenza, and he clearly pointed out that the adoption of the theory of bacillus with its chemical products, as an adequate and sufficient cause for all the phenomena of this protean malady, should be the foundation of all methods of prevention and cure. He thought that in any future epidemics much might be expected from the destruction of discharges and the intra-tracheal injection of antiseptic drugs.

*The Address in Surgery.*—If Mr. Greig Smith thought of the 1863 meeting of the Association, when four general addresses were given by four Bristol men, either he must have come to the conclusion that the fashion of choosing these orators had changed, or, unless he carried self-effacement to an unusual degree, he must have felt more than an ordinary pride that he was the only Bristol man to whom the honour of delivering one of these addresses had been given. Whether local talent is less pronounced or not than it was thirty years ago, there can be no doubt that the craft of surgery in general and Bristol professional reputation in particular were safe in the keeping of the Professor of Surgery at our Medical School. It was most unfortunate that the large Victoria Room, which formed such an excellent place for the other addresses, was not available for the Address in Surgery. But the room chosen, the Lecture Hall of Victoria Chapel, did fairly well, although the confusion caused by the arrival of late comers and the noise in moving chairs somewhat disturbed the audience, and must have annoyed the orator at first. But when all were seated absolute silence prevailed, and scarcely any one left till the address was over. Some disappointment was felt by those who knew what a pioneer Mr. Greig Smith had been in many departments of surgery that he did not choose for his subject one in which he could have given free scope to his great critical faculties, and at the same time have displayed his strength in developing improvements and overcoming difficulties. But "The Art of the Surgeon" is a subject full of interest and importance, and it was treated from a new and original point of view. It was not its matter, however, or its powerful plea for improved methods in training surgeons to practice their art which constituted the strength of the address. It was the charm of the delivery, the noble choice of words and their combination into well-balanced sentences, the literary finish and polish which pervaded the whole of it that made it a delight to the large audience which had the good fortune to hear it. Of course there was in it much that was worth thinking over, and it is good for the practical surgeon to be reminded how much of his work is really an art. Few people will be inclined to disagree with Mr. Greig Smith's statement that the mechanical side of surgery should receive more attention in the training of the student. But when we come to the suggestion that the younger men should operate under the immediate instruction of their seniors, then the difficulties are great, and it would be more profitable to ponder over the comparatively easy task of improving the present cumbrous curriculum, with a view to developing the opportunities of students and resident hospital officers for acquiring that art and manual dexterity on which Mr. Greig Smith so rightly insists.

*The Address in Public Medicine.*—It is difficult to see why there should be an address on such a subject as Public Medicine, or, if there is one, why anybody should be expected to listen to it, when it would have just as much effect if merely sent to the *Journal* and printed.[1] Sir Charles Cameron's stock of Irish humour no doubt on this occasion somewhat relieved the deadly dulness of the oration, which must, as a matter of course, consist very largely of a citation of statistics. The address this year included many points of interest on which Sir Charles Cameron's own experience gave additional weight to his conclusions. In dealing with the relation of increased mortality to social status, he pointed out that the means for lessening the excessive death-rate of the very poor do not lie altogether in the domain of public medicine: they must also be worked out by the political economist, aided by those philanthropists whose resources enable them to gratify their benevolent instincts. It is to be regretted that Sir Charles, by insisting too much on the purifying power of proper filtration, has somewhat weakened the position of analysts who make a point of knowing all they can of the "surroundings" of a water before giving a responsible opinion as to its potability. The case of Bristol, with its unventilated sewers and its comparatively low enteric fever death-rate, was adduced as evidence to show that the assertion that enteric fever is the disease most likely to be produced by sewer emanations is not a complete statement of the complex problem to be solved. Taking a general view of the various statements in the address dealing with the relation, direct or indirect, of infective disease-prevalence to filth-pollution of air, earth, and water, there would appear to be little doubt that its distinguished author leans strongly to the opinion that most of the micro-organisms which cause disease in England may have a saprophytic stage in their life-history. This phase may be obscured by special facilities of spread and other conditions not at present fully understood, but that it exists to a far larger

[1] By the way, it is strange that although the *Journal* gave us editorials on the President's Address and on the Addresses in Medicine and Surgery, it did nothing of the sort on the Address in Public Medicine.

extent than has as yet been recognised is by no means the least valuable suggestion in this address, supposing this interpretation of the author's attitude to be correct.

### THE SECTIONS.

For a full list of the readers and speakers in the various sections the reader must consult the copious details of the *British Medical Journal.* Here it is proposed only to sum up some of the more practical work done at the meeting.

*Medicine.*—This section was held in the Library of the Medical School, which gave ample accommodation to the large number of members attending it. Dr. Frederick Roberts, the President, began the proceedings with a most excellent address, in which he indicated the lines on which the section would work. Dr. Douglas Powell opened a discussion on "Functional Diseases of the Heart," with an able paper illustrated by a number of valuable tables. He treated exhaustively, both from the physiological and clinical standpoints, the various forms of functional heart-trouble. In the subsequent discussion, owing to the wide extent of the subject, the speakers dwelt each with some different form of heart-disturbance, rather than with one aspect of the whole group. On the whole, stress was laid on the neurotic rather than the mechanical theory of these complaints as the correct one. The discussion was well maintained for more than two hours, and was listened to by about ten lady doctors, whom the President invited to join in the discussion, on the plea that they should be able to offer special instruction on affections of the heart. He summed up the discussion by remarking that after the excitement, feasting, and smoking of the week, many might have some personal experience of the disorder. Dr. Sansom followed with a very interesting paper on the disorders of the heart subsequent to influenza, which also elicited a good discussion. Other papers dealing with various aspects of heart-disease were read. Dr. Harry Campbell and Dr. F. J. Wethered both read papers on the treatment of heart-disease by mechanical means (gymnastic movements). Dr. Wethered dwelt especially on this mode of treatment, combined with baths, as carried out at Nauheim by Drs. Schott; and he gave a very interesting practical demonstration of these exercises in a case of dilated heart.

On the second day the attendance was again large, to listen to a discussion on "Pyrexia and its Treatment." Dr. Hale White opened this in a paper which was a model of clearness, precision, scientific accuracy, and thoroughness. He gave an interesting account of the experimental work on the questions of production and loss of heat, and thermotaxis, and explained its application to every-day clinical experience. In the question of treatment, he showed how the specifics—such as salicylic acid in rheumatism and quinine in malaria—for those diseases in which we have them lower the temperature, not by acting directly upon it, but by attacking the specific poison of the disease. In other maladies he advised the external application of cold where it is necessary to directly treat the fever; and he deprecated the use of modern antipyretic drugs, as harmful to the patient, and dangerous from their analgesic and antipyretic properties, because they are liable to prevent the possibility of accurate diagnosis. Dr. Hale White's views were confirmed by subsequent speakers, with the exception of Dr. Shingleton Smith, who, whilst acknowledging the many virtues of the bath, thought that antipyretic drugs, especially phenacetin, might often be substituted for it with advantage. Dr. Osler, of Baltimore, was especially emphatic on the reduction of the mortality in cases of enteric fever by the systematic use of cold baths. The result of the discussion was very definite. The general opinion was: (1) Against the treatment of fever as fever unless clearly necessary, as such treatment tended to the mistake of treating the symptom rather than the disease; (2) in hyperpyrexia, to rely on the use of cold baths; (3) when necessary, to use the same means for the reduction of degrees of fever below hyperpyrexia; and (4) to abstain from the administration of antipyrine, antifebrin, and phenacetin. In addition, the value of quinine, under certain conditions, as an antipyretic, was fully recognised. The section had enough of water this day, as three papers dealing with climate and bath treatment were then read.

On the third day a good audience again assembled to hear the discussion on Ataxia, introduced by Dr. J. A. Ormerod, who read a most lucid and instructive paper on the subject. He gave a masterly account of the physiology and pathology of the different forms of ataxy, a most difficult subject to treat without obscurity. The sensory theory of ataxy was the one to which he inclined. Then followed a review of the clinical varieties of ataxy, with their distinguishing characteristics and modes of origin. Dr. Ormerod's paper was thoroughly appreciated, and the discussion which followed was well maintained until the end of the time allotted for sectional proceedings. Dr. Lloyd Andriezen remarked on some of the features of ataxy as presented amongst the insane, and Dr. Dawson Williams as it occurs in transient forms after the acute fevers. Dr. Waldo showed a case of tabes with remarkable arthropathies, and Dr. Michell Clarke exhibited another with peculiar features, and two cases of Friedreich's disease, in one of which the knee-jerks were preserved. He also showed sections of the cord from a fatal case.

The work done by the section was very satisfactory. Keen interest, and at times, enthusiasm, was maintained to the very end, and the successful result was largely due to the able manner in which the business was conducted by the President.

*Surgery.*—The meetings of the Surgical Section were held in the Physical Science Lecture Room of University College. On the first day there was a discussion on "The Operative Treatment of Perforative Ulcer of the Stomach and Intestines," introduced by Mr. Pearce Gould, who gave due credit to the suggestion of operation, which was given by Mr. Dobson in the *Bristol Medico-Chirurgical Journal* for December, 1883. Indeed, it appeared that Mr. Dobson had anticipated the surgery of to-day, for, beyond questions of *technique*, which can be known only by experience, there has been in the cases subjected to operation simply a fulfilling of his surgical foresight.

On the second day the subject for discussion was one of more recent development. Mr. William Thorburn, in bringing before the section "The Surgical Treatment of Injuries of the Spine and Spinal Cord," ably advocated the adoption of an expectant attitude in almost all cases of injuries of this class. He showed clearly that operation was not likely to be of service except in injuries of the spinous processes and laminæ, in hemorrhage, in compound fractures, in late meningitis, and in lesions of the cauda equina. It is in the last of these conditions that operation is especially useful. The reason for the adoption of this conservatism lies in the fact that there is practically no evidence of any power of physiological recovery in a badly crushed cord, even if the pressure be relieved, whether such relief occur by diastasis at the time of the injury or is gained subsequently by the operation of laminectomy. Subsequent speakers were, on the whole, inclined to endorse Mr. Thorburn's views.

The third day was reserved for a discussion on "The Treatment of Injuries of the Lower End of the Humerus." This was introduced by Mr. Jonathan Hutchinson, jun., who advocated passive movements as soon as union was at all firm. The attendance on this day was not so large as on the other days. Either the subject for discussion was less attractive, or members were suffering so much from the results of excessive hospitality, that they could not attend so early as 9.30.

The discussions were distinctly of a high order, and it may be said that the Science and Art of Surgery have been advanced by them. The general tone of discussions and papers was one of caution; and the prevailing opinion seemed to be that in operative procedure surgery had been going quite fast enough, and that the time had arrived at which we might well pause and reflect for a season on what had been done, rather than seek for new fields of surgical enterprise.

The section was presided over in a very able manner by Mr. Mitchell Banks, who in a characteristically gentle and genial manner summed up the opposing views at the end of the various discussions and papers. The presidential duties were performed with much perspicacity and tact, such as are only found in a man of wide and general experience.

*Obstetric Medicine and Gynæcology.*—The proceedings in this section, which was held in Lecture Room, No. 2, University College, began with a short address by the President, Dr. J. G.

Swayne, who gave some interesting reminiscences of midwifery and medicine in Bristol. Dr. Robert Barnes then opened a discussion on "The Induction of Premature Labour for Diseases of the Mother not causing Obstruction to Delivery." His speech, as was expected, promised well for a good discussion; but, unfortunately, succeeding speakers did not address themselves strictly enough to the matter in hand. The general result of the discussion may be summarised thus: (1) That induction is necessary when the presence of the fœtus and the enlarged uterus endanger the life of the mother in cardiac disease, aneurism, persistent vomiting, eclamptic fits, or albuminuria; (2) that the introduction of a bougie, followed by dilatation or not, is the best method; (3) that dilatation should be reserved for those cases in which speed is necessary. There were, however, some noteworthy dissentients. Dr. Tweedy considered induction unnecessary and unjustifiable in albuminuria. He prefers to use hypodermic injections of morphia, which apparently causes the death of the fœtus and the cessation of the symptoms. Professor Byers took a somewhat similar view.

The second day's work opened with a discussion on "The Treatment of Hemorrhage during the last two months of Pregnancy." This was introduced by Dr. W. P. Smyly, who laid stress on the inadvisability of hurrying delivery in cases of placenta prævia, as he was in favour of leaving delivery to the natural efforts after version had been performed. He maintained that the easy dilatation of the os meant in many cases easy laceration. He pointed out that in accidental hemorrhage the effusion of blood ceased when the intra-uterine pressure became equal to the general blood-pressure, and so the best results might be looked for by plugging the cervix and vagina. He did not recommend forcible dilatation, because it added to the shock and depression. Most of the speakers in the long discussion which followed took the same view as the opener, but it was pointed out that measures which were suitable and safe in a hospital were not always the best for cases where skilled assistance and supervision were not ready to hand, and that, therefore, the expectant plan was not to be recommended in cases of hemorrhage occurring in ordinary practice.

The third day was occupied by the reading of several papers. Dr. C. E. Purslow narrated "Four Cases in which Separation of the After-coming Head had occurred during Delivery." He dwelt, in a practical manner, upon the well-known difficulties in extracting a head under such circumstances. Dr. C. J. Cullingworth gave an account of "A Case of Extra-uterine Gestation, in which a Living Child was removed, the Placenta left undisturbed, and the Abdominal Wound entirely closed." The case proved fatal about six weeks after operation. The paper was discussed at some length, and it was pointed out that the absorption of the placenta is much slower than was supposed. Dr. Rabagliati's paper on "Some Obscure Ailments which simulate Ovarian Disease," was full of interest, but it was too long to be read in full.

The section was well attended throughout, and the interest in the work was thoroughly maintained. There was a large contingent of ladies, some of whom took part in the discussion, apparently without causing the inconvenience which was apprehended by some of the gentlemen.

*Public Medicine.*—Professor Corfield's presidential address in this Section, which met in the Lecture Theatre of the Museum and Library, dealt entirely with the Vital Statistics of 1893. The enormous increase of the death-rate from diphtheria in some of the large towns came in for special comment, while the decrease in others of the large towns was instanced as pointing to the necessity for a careful study of the conditions of those towns in which the disease is decreasing, so that we may be better able to understand why it is increasing in many others. Diphtheria came in for a good share of attention in the proceedings. Dr. Slade King urged the necessity for the isolation of patients for a period of three weeks or a month after they were supposed to be cured. Further emphasis was given to this point by the communication made to the Section by Dr. Hermann Biggs, of the New York Bacteriological Department, who stated that, as the result of systematic bacteriological examination of inoculated cultures from cases of diphtheria, embracing as many as 6,000 primary and an equal number of secondary examinations, it was found that the convalescent cases assisted to a large extent in the dissemination of the disease. For long periods after the disease had entirely disappeared, when the patient was perfectly well in appearance and mingling with other people, there were still virulent organisms in the throat. These remained from two days up to seven weeks after the disappearance of the membrane, and, upon being tested by experimentation, were proved to be as virulent as at the height of the disease. The New York arrangements for the examination of cultures of diphtheria are remarkably complete. "Culture outfits," with explicit directions for their use, are obtainable at 40 stations. Cultures made by the private attendant are examined by the department, and reports forwarded after twelve hours' observation. In spite of the criticism that the bacteriological examination of cultures prepared by practitioners with little experience in biological methods, is liable to much chance of error, Dr. Biggs declared that the trustworthiness of the examination was marked, and that errors were very few. The system is certainly worthy of attention by the authorities of those districts in which the increase of diphtheria is a cause for anxiety.

Following the discussion on papers by Mr. E. Stephens and Sir Henry Thompson on Cremation, a strong resolution was unanimously passed urging the necessity for a reform in the procedure for the certifying of deaths. The gradual but progressive favour with which cremation is coming to be regarded, especially for use in infectious diseases, is evidenced by the recent erection of crematoria at various places. In the Bristol Channel, the crematorium erected on the Flat Holm by the Cardiff Port Authority for use in case of cholera is available for the Authorities of Bristol and Gloucester in case of need.

Dr. Arnold Evans made out a strong case for the aerial convection of small-pox, and the experience at Bradford showed that the diffusion corresponded with the prevailing winds. The paper contributed by Dr. J. Priestley in illustration of the recent Leicester small-pox experience, drew forcible attention to the sacrifice of unprotected children accumulating in that town. The statistics showed with terrible emphasis the suffering and the mortality which are the fate of the unvaccinated.

